# Role of non-classical renin-angiotensin system axis in renal fibrosis

**DOI:** 10.3389/fphys.2015.00117

**Published:** 2015-04-21

**Authors:** Lin-Li Lv, Bi-Cheng Liu

**Affiliations:** Institute of Nephrology, Department of Affiliated Zhongda Hospital, Southeast UniversityNanjing, China

**Keywords:** renin-angiotensin system, renal fibrosis, angiotensin-converting enzyme 2, (pro)renin, angiotensin(1–7)

## Abstract

The renin–angiotensin system (RAS) is a major regulator of renal fibrosis. Besides the classical renin/Angiotensin-converting enzyme (ACE)/angiotensin II (Ang II)/AT1 and AT2 axis, multiple new axes have been recently described. The new members have added new dimensions to RAS, including the ACE2/Ang(1–7)/Mas receptor axis, the prorenin/(pro)renin receptor(PRR)/intracelluar pathway axis, and the Angiotensin A (Ang A), alamandine-Mas-related G protein coupled receptor D(MrgD) axis. This review summarized recent studies regarding role of the non-classical RAS axis in renal fibrosis, and its possible implications to the intervention of progression of chronic kidney disease.

## Introduction

Glomerulosclerosis and tubular interstitial fibrosis are the final common manifestation of a wide variety of chronic kidney diseases (Liu, 2006). One common feature of progressive renal disease is the proliferation of resident renal cells, excessive accumulation and deposition of extracellular matrix (ECM), and tissue retraction (Eddy, 2014). The renin–angiotensin system (RAS) is a major regulator of renal fibrosis (da Silveira et al., 2010). Both tissue and circulatory RAS are believed to be overactivated in the development of renal fibrosis (Navar, 2014). Tremendous studies have demonstrated that angiotensin II (AngII) play a powerful role in renal fibrosis by mediating the release of transforming growth factor-β (TGF-β) and activating inflammatory process (Mezzano et al., 2001). Moreover, our previous studies have suggested connective tissue growth factor (CTGF)/integrin-linked kinase (ILK) participated in the renal fibrosis development mediated by AngII (Liu et al., 2007). We also proposed the interaction of AngII and inflammation might be the critical node in pathogenic glomerulotubular feedback loop (Zhang and Liu, 2011). In addition to angiotensin-converting enzyme (ACE) dependent AngII generating system, chymase as an alternative AngII generating enzyme, was involved in the development of diabetic/hypertensive nephropathy (Huang et al., 2003). However, in recent years, RAS is far more complex than originally described (Chappell, 2012). New members have been added to the RAS family, which played critical roles in the process of renal diseases (Chappell, 2012; Simoes e Silva et al., 2013). This review will focus on the role of new RAS members and the mechanism in promoting glomerulosclerosis and tubularinterstitial fibrosis.

## ACE2/Ang(1–7)/Mas receptor axis

Angiotensin-converting enzyme 2 (ACE2) is a monocarboxypeptidase. ACE2 converts angiotensin II (Ang II) to angiotensin 1–7 [Ang(1–7)], which binds to Mas (Vickers et al., 2002). ACE2 shares 40–42% homology with ACE but shows different biochemical activities (Donoghue et al., 2000). ACE and ACE2 co-localize in the brush border of mouse proximal tubules (Ye et al., 2006). However, they localize to different cell types in glomeruli. Within the glomerulus, ACE2 is present in glomerular epithelial cells and glomerular mesangial cells. Glomerular ACE is mainly expressed in endothelial cells. In contrast to the fibrogenic and proliferative actions of the ACE/Ang II/AT1 axis, it has been suggested that the ACE2/Ang(1–7)/Mas axis exerts anti-fibrogenic and anti-proliferative actions (Ferrario, 2011).

### ACE2 and renal fibrosis

Many studies have suggested a protective role for ACE2 in different models of renal damage or diseases, including subtotal nephrectomy (Dilauro et al., 2010), ischaemia/reperfusion kidney injury (da Silveira et al., 2010), unilateralureteral obstruction (UUO) (Liu et al., 2012). Acquired genetic ACE2 deficiency accelerates renal injury in those experimental models. Dilauro et al. reported that kidney ACE2 is downregulated in the early period after 5/6 nephrectomy (5/6 NX). Inhibition of ACE2 with MLN-4760 (MLN) in 5/6 NX mice increases albuminuria via an AT1 receptor dependent mechanism, independent of blood pressure (Dilauro et al., 2010). In studies from Zhong et al., wild-type mice were infused with Ang II and treated daily with recombinant human ACE2. It showed that ACE2 could attenuate Ang II-induced pressor response and normalize renal Ang II levels and oxidative stress. Importantly, ACE2 could prevent Ang II-induced tubulointerstitial fibrosis (Zhong et al., 2011). Regarding glomerularsclerosis, it has been shown that glomerular staining for fibronectin was increased in both db/db and db/m mice that were treated with ACE2 inhibitor (MLN-4760) compared with vehicle-treated group (Ye et al., 2006).

ACE and ACE2 activity play an important role to balance the expression of angiotensin II and angiotensin(1–7) (Chappel and Ferrario, 2006). The integral relationship of ACE/ACE2 is critically involved in process of renal fibrosis. Our previous study has suggested that treatment of HK-2 cells to bovine serum albumin (BSA) led to a significant dysregulation of ACE/ACE2 expression in time and concentration dependent manner (Liu et al., 2009). However, in a recent study with cultured podocytes, insulin increases ACE2 expression and favors an “anti-angiotensin II” regarding ACE/ACE2 gene expression balance and decreases fibronectin expression as a marker of fibrosis. In the presence of albumin, the effect of insulin on ACE2 disappeared. The authors suggest that albuminuria might block the beneficial effect of modulating RAS balance for preventing diabetic nephropathy (Marquez et al., 2014). Moreover, urinary albumin excretion (UAE) increased significantly in ACE2 inhibitor-treated group as compared with vehicle-treated db/db mice (Ye et al., 2006). These studies suggested the close correlation between albumin and ACE2. ACE2 reduction could accelerate albuminuria, while albumin in turn could disrupt the balance of ACE/ACE2. The interplay between ACE and ACE2 may govern the formation and metabolism of Ang effector peptides, the regulators that determines their balance need more studies (Brosnihan et al., 2005). Moreover, it is still unclear whether the protective effects of ACE2 is due to the reduction of AngII, the formation of Ang(1–7) or the increased ratio of Ang(1–7) to AngII.

Transforming growth factor-β1 (TGF-β1) is a key mediator in the process of renal fibrosis (Lan and Chung, 2012). The interaction between TGF-β and ACE2-Ang(1–7)-Mas has been observed. In NRK-52E cells, TGF-β1 decreases ACE2 and Mas and Ang(1–7) conversion from Ang II. The combination of Ang-(1–7) and Mas could attenuate TGF-β1 (but not high glucose)-induced fibronectin (Chou et al., 2013). Liu et al. showed that deletion of ACE2 resulted in increasing ratio of intrarenalAng II/Ang(1–7) and enhanced renal fibrosis in the UUO nephropathy. These changes were attributed to the increase in the intrarenalAng II signaling (AT1-ERK1/2 mitogen-activated protein kinase), TGF-β/Smad2/3, and NF-κB signaling pathways. The authors concluded that enhanced AngII-mediated TGF-β/Smad and NF-κB signaling might be one of the mechanisms by which loss of ACE2 enhances renal fibrosis and inflammation (Liu et al., 2012).

### Ang(1–7) and renal fibrosis

Multiple small peptides can be derived from degradation of AngII and have been demonstrated to have local physiological effects in the kidney. Those peptides include Ang(1–7) (Alzayadneh and Chappell, 2014), Ang(2–8) (Kemp et al., 2012), Ang(3–8) (Chai et al., 2004), Ang(3–4) with Ang(1-5), and Ang(1–4) as intermediate peptides (Axelband et al., 2009). Among those peptides, Ang(1–7) is a biologically active heptapeptide from the degradation of angiotensin II by ACE2, the role of which in kidney disease have been studied most extensively (Ferrao et al., 2014). Santos et al. firstly found a G protein-coupled receptor for Ang(1–7), the Mas receptor. It provides the molecular basis for the physiological actions of this peptide (Santos et al., 2003). Although there is now much evidence that Ang(1–7) may exert a protective role in experimental models of renal diseases, a few studies have revealed controversial results. Consequently, the role of Ang(1–7) in the progression of CKD requires further clarification, especially its therapeutic role in different types of kidney diseases.

Ang(1–7) has been demonstrated to be able to ameliorate streptozotocin induced diabetic renal injury. In mouse model of streptozotocin induced diabetic nephropathy, large dose of Ang(1–7) improved renal function, attenuated glomeruli sclerosis, oxidative stress, and cell proliferation (Zhang et al., 2015). Moreover, the effects of large-dose Ang(1–7) alone and in combination with valsartan were superior to valsartan alone, but the combination had no significant synergistic effect compared with Ang(1–7) alone (Zhang et al., 2015). Mori et al. administered Ang(1–7) or saline to 5-mo-old db/db mice. The treatment reduced kidney weight and ameliorated mesangial expansion and increased urinary albumin excretion. The protective effect on renal fibrosis was correlated with dephosphorylation of the signal transducer and activator of transcription 3 (STAT3) pathway (Mori et al., 2014).

In contrast, another study using a moderate dose of Ang(1–7) to treat diabetic nephropathy showed no benefitial effect in reducing the levels of serum creatinine and creatinine clearance (Singh et al., 2010). Shao et al. reported that treatment of STZ induced diabetic rat by constant Ang(1–7) vein injection for 6 weeks, renal function was found to be even worse than diabetic rats (Shao et al., 2008). In the 5/6 NX mouse model of chronic kidney disease (CKD), treatment of 5/6 NX mice with Ang(1–7) increased kidney and plasma levels of Ang(1–7) but did not change levels of blood pressure, urinary albumin excretion (Dilauro et al., 2010).

The underlying mechanism for Ang(1–7)'s role in renal fibrosis needs to be further clarified before the therapeutic role to be determined. A few studies have explored the molecular mechanisms of the renoprotection induced by Ang(1–7). Alzayadneh et al. found that Ang(1–7) could abolish Advanced glycation end products (AGEs)-induced activation of the MAP kinase ERK1/2 to a similar extent as the TGF-β receptor kinase inhibitor (Alzayadneh and Chappell, 2014). Ang(1–7) could also decrease the expression of collagen IV, TGF-β1, VEGF, NOX4, p47phox, PKCα, and PKCβ1, and the phosphorylation of Smad3 (Zhang et al., 2015). AGEs exposure in NRK-52E renal epithelial cells for 48 h significantly reduced the intracellular levels of Ang(1–7) approximately 50%. Treatment with Ang(1–7) could reverse AGEs-induced cellular hypertrophy and myofibroblast transition (Alzayadneh and Chappell, 2014). Hyperlipidemia is an independent risk factor for renal disease, and lipid deposition is closely correlated with glomerulosclerosis. Huang et al. found that Ang(1–7) could inhibit low-density lipoprotein accumulation and decreases cholesterol levels via modulating the low-density lipoprotein receptor (LDLr) -sterol regulatory element-binding protein (SREBP)-cleavage activating protein (SCAP) negative feedback system through the Mas receptor (Shao et al., 2008). Interestingly, Ang(1–7) antagonize the effect of Ang II on lipid accumulation. Our previous studies showed that Ang II increased lipid droplet accumulation in human renal mesangial cells (HMCs) via enhanced translocation of the SCAP/SREBP-2 complex from the endoplasmic reticulum (ER) to the Golgi in HMCs (Ma et al., 2013). Since the close correlation between renal lipid accumulation and glomerulosclerosis, tubulointerstitial fibrosis, especially in diabetic kidney disease has been shown, we thought that Ang(1–7) might prevent the progression of renal fibrosis through its inhibition on lipid accumulation (Wang et al., 2005). This could be a interesting aspect in exploring the therapeutic role of Ang(1–7) in treating renal fibrosis.

Consequently, to address the theraputic potential of Ang(1–7), the related issues that need to be clarified include: (1) the effect of different doses of Ang(1–7); (2) the effect of its combination with an angiotensin receptor blocker; (3) the mechanism by which it exerts the protective effect.

## Prorenin/(pro)renin receptor (PRR)/intracellular signaling axis and renal fibrosis

A new axis in the RAS research field provoking much interest recent years is the prorenin/PRR/intracelluar pathway axis. Renin is synthesized in juxtaglomerular cells of the afferent arterioles of the kidney as preprorenin. Prorenin was generated by cleavage of a 23 amino acid peptide at carboxyl terminus of preprorenin. Prorenin is activated to renin by cleavage of 43-amino acid N-terminal prosegment by proteases (Song and Yosypiv, 2011). The receptor binding renin and prorenin, named the PRR, was cloned in 2002 (Nguyen et al., 2002). The binding induces prorenin enzymatical activation without cleavage of the prosegment, and triggers intracellular signaling pathways independent of the RAS. Through activating multiple intracellular pathways, prorenin/PRR participate in the development of renal fibrosis independent of ANG II. Recent findings have found additional role of PRR, participating in the functions of vacuolar proton ATPase and constituting the Wnt receptor complex (Oshima et al., 2014). The novel biological effect of PRR is that it regulated several cellular homeostatic processes including autophagy which was described in a recent review (Binger and Muller, 2013).

### Prorenin/PRR in resident kidney cells

In multiple types of resident kidney cells, it has been shown that prorenin/PRR could activate several intercellular signaling pathways leading to renal fibrosis. In the human kidney, confocal microscopy has identified that the PRR localized in mesangium of glomeruli and in the subendothelium of coronary and kidney artery (Nguyen et al., 2002). However, a number of studies showed predominant expression of PRR in the collecting duct, particularly on the apical membrane of intercalated cells (Advani et al., 2009; Gonzalez et al., 2013). Thus, the cellular localization of PRR in addition to its molecular form is intriguing. The protein is shown to accumulate intracellular or in plasma membrane or in the trans-Golgi. Indeed, Prieto et al. found that hyperglycemia induces PRR trafficking alterations and increase PRR abundance in plasma membrane (PM) in the collecting duct. Recent evidence demonstrated that PRR is predominantly activated by AngII, which is mediated by (cycloxygenase-2) COX-2/E-prostanoid4 pathway (Wang et al., 2014a,b).

In renal mesangial cells, Zhang et al. showed that elevated prorenin could be activated through receptor binding without being proteolytically converted to renin. This activation leads to increased expression of PAI-1 and transforming growth factor-β1 via AngII-independent and AngII-dependent mechanisms (Zhang et al., 2012). Melnyk et al. also showed that two distinct pathways were activated by renin and prorenin, a TGF-dependent pathway and a TGF-independent pathway (Melnyk et al., 2009). Additionally, high glucose exposure enhanced TGFβ1-CTGF signaling cascade through PRR (Huang et al., 2011). In renal epithelial cell, Saito et al. demonstrated that indoxyl sulfate, which is a uremic toxin, could induce expression of PRR and prorenin. PRR knock-down inhibited indoxyl sulfate-induced expression of TGF-β1 and α-smooth muscle actin in HK-2 cells. The up-regulation of prorenin expression and activation of PRR was related with reactive oxygen species and activation of Stat3 and nuclear factor-κB (Saito et al., 2014). In human embryonic kidney cells (HEK), both renin and prorenin stimulate TGF-β, fibronectin and PAI-1 expression via a Nox4 dependent mechanism. The study indicates a PRR/Nox4 pathway in the development of kidney fibrosis through the generation of superoxide anions (Clavreul et al., 2011).

Thus, prorenin and PRR participate to an overall switch toward a pro-fibrotic state of the kidney cells, including renal mesangial cells, proximal tubular cells and embryonic kidney cells. Prorenin/PRR could induce multiple pathways of intercellular signaling via AngII-independent mechanism, leading to the development of renal fibrosis (Ichihara et al., 2006a).

### Prorenin/PRR in animal models

Activation of PRR by prorenin have been implicated in the development and progression of renal fibrosis in animal models. Kaneshiro et al. showed that in (pro)renin receptor-transgenic rat, proteinuria and significant glomerulosclerosis was developed. In kidney, mitogen-activated protein kinases (MAPK) were activated and expression of TGF-β1 was enhanced, and these changes were AngII-independent (Kaneshiro et al., 2007). Ichihara et al. showed that in AT1a receptor-deficient (ATKO) mice, prorenin/PRR/MAP kinases ERK1/2 axis plays a pivotal role in the development of diabetic nephropathy. Treatment of the diabetic WT or ATKO mice with ACE inhibitor failed to prevent the diabetic nephropathy completely, while chronic infusion of mouse HR decoy peptide (HRP) (HRP inhibits prorenin binding to PRR and non-proteolytic activation) (Ichihara et al., 2004) completely prevented the increase in urinary protein excretion and the development of glomerulosclerosis (Ichihara et al., 2006b). In spontaneously hypertensive rats (SHR), inhibition of non-proteolytic activation of prorenin by HRP decreased renal AngII levels and attenuated the development and progression of proteinuria and glomerulosclerosis, the effect is independent of circulating RAS or arterial pressure (Ichihara et al., 2006a). These findings suggest that prorenin/PRR may induce renal fibrosis through multiple intracellular signaling, either alone or in concert with activation of renal tissue RAS.

However, a recent study indicated that increased expression of PRR is not sufficient to induce renal injury. Rosendahl et al. generated a mouse that constitutively overexpressed PRR, they found no difference in systolic blood pressure or albuminuria between wild type and PRR overexpressing mice. And no renal or cardiac fibrosis was detected with histological examination in mutant mice. The authors suggest that PRR might not play central role in organ damage *per se* (Rosendahl et al., 2014). Interestingly, transgenic overexpression of the prorenin in rats does not cause renal fibrosis, whereas leads to myocardial hypertrophy, proteinuria, and hypertension (Peters et al., 2008). Nguyen et al. proposed in a well summarized review on PRR, that prorenin may be an amplifier of fibrosis, in addition to high glucose, inflammation, or immunological cytokines (Nguyen and Muller, 2010).

Moreover, recent studies suggest that PRR is not always devil to kidney. Oshima et al. reported that PRR might be necessary for the maintenance of normal podocyte structure and function. Deficiency of the PRR in murine podocytes resulted in disruption of the filtration barrier and accumulation of intracellular vesicles (Oshima et al., 2011). More studies are needed to determine the contribution of prorenin/PRR in renal fibrosis.

## Other new members of RAS:angiotensin A (Ang A), alamandine-Mas-related G protein coupled receptor D(Mrgd)

Jankowski et al. first discovered a novel human Ang-derived peptide, Ang A (Ala-Arg-Val-Tyr-Ile-His-Pro-Phe) with increased level in end-stage renal failure patients compared with healthy humans (Jankowski et al., 2007). However, Yang et al. demonstrated that Ang A displays similar *in vitro* and *in vivo* properties as AngII, the effect of this peptide is mediated via AT1a receptors. Ang A might be not a naturally occurring peptide that modulates the pressor and renal hemodynamic effects of AngII (Jankowski et al., 2007). Additional studies are needed to characterize the role of this peptide in the kidney function and renal fibrosis.

By using mass spectrometry, Lautner et al. found a new member of RAS, Alamandine. Alamandine is formed from catalytic hydrolysis of the angiotensin A by human ACE2. The peptide binds to Mas-related G protein coupled receptor D(MrgD), the effects include vasorelaxation, blunting of isoproterenol-induced heart fibrosis, and anti-hypertensive action in SHR (Lautner et al., 2013). Alamandine is an endogenous peptide of heart tissue and also presents in human blood (Lautner et al., 2013). Lautner et al. revealed that nephropathic patients have increased plasmatic concentration of alamandine. Administration of alamandine produced a long-term antihypertensive effect and decrease of collagen I, III, and fibronectin accumulation in the heart (Lautner et al., 2013). However, the role of these novel RAS components in renal fibrosis is still lacking investigation.

## Can we block/activate new member of RAS to ameliorate renal fibrosis?

XNT(1-[(2-dimethylamino)ethylamino]-4-(hydroxymethyl)-7-[(4-methylphenyl) sulfonyl oxy]-9H-xanthene-9-one) is a recently described synthetic activator of ACE2 (Hernandez Prada et al., 2008). It has been reported to exert various organ-protective effects, which are attributed to the activation of ACE2. XNT could attenuate thrombus formation, control pulmonary hypertension, reverse cardiac fibrosis (Ferreira et al., 2009). Administration of XNT to SHR resulted in the decrease in blood pressure, which was also associated with improvements in renal fibrosis (Hernandez Prada et al., 2008). However, a recent study reported by Haber et al. showed that neither plasma nor kidney ACE2 activity in Ang II infusion animal model was affected by XNT. *In vitro* and *in vivo* experiments confirmed a lack of enhancement of ACE2 enzymatic activity by XNT. They concluded that the biological effects of this compound are ACE2-independent (Haber et al., 2014). Studies on the therapeutic effect of XNT in renal fibrosis are still very limited. More observations are needed to prove its potential in treating renal fibrosis.

Current anti-RAS therapy only has limited efficiency, partly because of compensatory upregulation of renin expression. Handle region peptide (HRP) mimics part of the prosegment of prorenin and inhibits prorenin binding to PRR and non-proteolytic activation (Suzuki et al., 2003). It was originally considered as a PRR antagonist, which evokes much interest. However, its therapeutic effect *in vivo* was still in controversial. HRP has been shown to prevent glomerulosclerosis in diabetic nephropathy (Ichihara et al., 2004). Afterwards, other *in vivo* studies have shown no therapeutic effects, which arise skepticism (Muller et al., 2008). Importantly, it is still unclear to what degree PRR blockade adds benefits in combination with classic RAS blockade. A recent study showed that in diabetic TGR (mREN2)27 rats, HRP given on top of aliskiren (a direct inhibitor of human renin), did not alter the effects of aliskiren on blood pressure, RAS activity, or aldosterone. However, it counteracted the beneficial effects of aliskiren in the kidney (te Riet et al., 2014). In SHR, it also showed that HRP counteracted the beneficial effects of aliskiren on blood pressure, coronary function, and cardiac hypertrophy in an angiotensin-independent manner (van Esch et al., 2011). Thus, the therapeutic effect of HRP for renal fibrosis is still in controversial which need more observation.

Ang(1–7)/Mas receptor axis plays a countermeasure role to that of angiotensin II/AT1 receptors. Ang(1–7)/Mas receptor might be a potential target for developing oral active agonist treating renal fibrosis. Wiemer et al. firstly described a non-peptide mimic of Ang(1–7), compound AVE 0991 which efficiently mimics the effects of Ang(1–7) on the endothelium (Wiemer et al., 2002). Suski et al. showed that Ang(1–7) peptidomimetic AVE0991 partially reversed atherosclerosis-related changes in apoE(-/-) mice (Suski et al., 2013). In adriamycin (ADR)-induced nephropathy, treatment with AVE 0991 improved renal function and attenuated histological changes (da Silveira et al., 2010). Ang(1–7) mimic peptide might hold a promise in future, but it need more investigations in multiple models of kidney injury.

Since the unsatisfied effect of RAS blockade on controlling renal fibrosis, recently, Zhou et al. aimed to find a treatment strategy to simultaneously target multiple RAS genes. They found that overexpression of either *β*-catenin or Wnt ligands induced the expression of all RAS genes. Interestingly, a small-molecule-weight *β*-catenin inhibitor ICG-001 abolished RAS activation. Meanwhile, ICG-001 therapy restored expression of nephrin, podocin, and Wilms' tumor 1, attenuated interstitial myofibroblast activation, repressed matrix expression, and inhibited renal inflammation and fibrosis. The results indicated that blockade of Wnt/β-catenin signaling can simultaneously repress multiple RAS genes, thereby leading to the reversal of established proteinuria and kidney injury (Zhou et al., 2015).

Further research on the contribution of those novel axes to renal fibrosis is in warranted. The clarification of the molecular mechanism mediating renal fibrosis may lead to the development of new approaches in the design of agonist or antagonists of RAS axis.

## Summary

**Figure 1:**
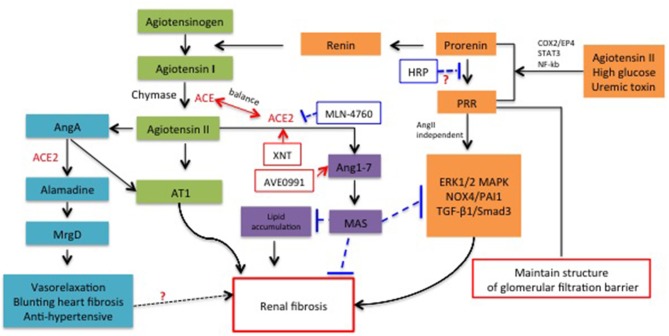
**Schematic representation for the role of new members of RAS system in renal fibrosis and the potential molecules targeted RAS for therapeutic application**.

In addition to classical renin/ACE/Ang II/AT1 and AT2 axis, recent studies have shown that multiple new members of RAS system play critical role in both glomerularsclerosis and tubularsclerosis, with synergic or antagonistic effect to classical RAS (summarized in Figure 1). However, the exact mechanisms through which the new players contribute to renal fibrosis are still unclear and the therapeutic potential targeting those members are still in controversial. Thus, based on the review mentioned above, the issues that need further observations include: How did the microenvironment (lipid accumulation, albumin, inflammation etc.) change the ACE/ACE2 axis balance and how to regulate it properly? How did the PRR been switch to pro-fibrotic factor and what is the exact role in development of renal fibrosis? What is the role of Ang A, alamandine-MrgD in renal fibrosis? Shall we develop more reliable approaches to prevent or reverse renal fibrosis with agonist or antagonists of new RAS members? Obviously, understanding these issues will provide more strategies for the intervention of renal fibrosis.

### Conflict of interest statement

The authors declare that the research was conducted in the absence of any commercial or financial relationships that could be construed as a potential conflict of interest.
